# Truncating Variants Contribute to Hearing Loss and Severe Retinopathy in *USH2A*-Associated Retinitis Pigmentosa in Japanese Patients

**DOI:** 10.3390/ijms21217817

**Published:** 2020-10-22

**Authors:** Akira Inaba, Akiko Maeda, Akiko Yoshida, Kanako Kawai, Yasuhiko Hirami, Yasuo Kurimoto, Shinji Kosugi, Masayo Takahashi

**Affiliations:** 1 Department of Ophthalmology, Kobe City Eye Hospital, Kobe, Hyogo 650-0047, Japan; inaba.akira.32s@st.kyoto-u.ac.jp (A.I.); akiko.yoshida.fe@riken.jp (A.Y.); kanako.kawai@riken.jp (K.K.); yhirami@kcho.jp (Y.H.); ykurimoto@mac.com (Y.K.); retinalab@ml.riken.jp (M.T.); 2 Laboratory for Retinal Regeneration, RIKEN, Center for Biosystems Dynamics Research, Kobe, Hyogo 650-0047, Japan; 3 Department of Medical Ethics, Graduate School of Medicine, Kyoto University, Kyoto 606-8501, Japan; kosugi@kuhp.kyoto-u.ac.jp

**Keywords:** retinitis pigmentosa, Usher syndrome, *USH2A*, inherited retinal degeneration, clinical sequence

## Abstract

*USH2A* is a common causal gene of retinitis pigmentosa (RP), a progressive blinding disease due to retinal degeneration. Genetic alterations in *USH2A* can lead to two types of RP, non-syndromic and syndromic RP, which is called Usher syndrome, with impairments of vision and hearing. The complexity of the genotype–phenotype correlation in *USH2A*-associated RP (*USH2A*-RP) has been reported. Genetic and clinical characterization of *USH2A*-RP has not been performed in Japanese patients. In this study, genetic analyses were performed using targeted panel sequencing in 525 Japanese RP patients. Pathogenic variants of *USH2A* were identified in 36 of 525 (6.9%) patients and genetic features of *USH2A*-RP were characterized. Among 36 patients with *USH2A*-RP, 11 patients had syndromic RP with congenital hearing problems. Amino acid changes due to *USH2A* alterations were similarly located throughout entire regions of the *USH2A* protein structure in non-syndromic and syndromic RP cases. Notably, truncating variants were detected in all syndromic patients with a more severe retinal phenotype as compared to non-syndromic RP cases. Taken together, truncating variants could contribute to more serious functional and tissue damages in Japanese patients, suggesting important roles for truncating mutations in the pathogenesis of syndromic *USH2A*-RP.

## 1. Introduction

Retinitis pigmentosa (RP) is the most common type of inherited retinal degenerative disease (IRD) and is clinically and genetically heterogeneous. Symptoms of this disease include night blindness, visual field constriction and a decline in vision. More than 70 and 270 causal genes in RP and in IRD, respectively, have been reported by the University of Texas Houston Health Science Center, Houston, TX, USA., (https://sph.uth.edu/retnet/). Although there are racial differences in causal genes, *USH2A* is one of the most frequent genes in Caucasian, Japanese and other populations [1,2]. Alterations in *USH2A* are responsible for Usher syndrome, which is the most common syndromic RP with sensorineural hearing loss [3]. Usher syndrome is classified into three types, type I, II and III, and causal genes of type II include *USH2A, ADGRV1*, and *WHRN* (*DFNB31*) [4]. *USH2A* is responsible for about 80–90% of Usher syndrome type II and *USH2A*-causing Usher syndrome is called Usher syndrome type IIa, which is an autosomal recessive disease [5,6,7]. In addition, non-syndromic RP and non-syndromic hearing loss can be also caused by *USH2A* variants [8,9,10]. Alterations in *USH2A* lead to a wide range of phenotypes and severity of the diseases.

*USH2A* is located on chromosome 1q41 in the human genome and is approximately 790 kb of genomic DNA containing 72 exons [11]. This gene codes for the transmembrane protein USH2A (Usherin) which is expressed in the junction between the inner and outer segments, the cilia region in the photoreceptor cells [12,13,14]. USH2A plays important roles in the development and homeostasis of the inner ear and the retina [15,16]. Because this protein belongs to cilial proteins, *USH2A*-associated diseases are categorized to ciliopathies [17,18]. In previous studies, *USH2A* is a frequent causal gene in Japanese RP patients as well as Caucasian RP patients, yet the mutation spectrum is different among different ethnic groups [19,20]. Genotype–phenotype correlation in *USH2A* has been reported where truncating variants are associated with more severe visual and hearing impairments [9,21,22], and similar trends have also been reported in Asians [23,24]. In contrast, there are studies reporting that the differences in phenotypes are not clear in syndromic and non-syndromic or truncating variants and non-truncating variants [25,26]. Furthermore, there are few reports in Japanese patients examining the genotype–phenotype correlation in *USH2A*-RP.

In this study, we investigated the genetic and clinical characteristics focusing on the relationship between gene alterations and syndromic or non-syndromic *USH2A*-RP in Japanese patients. This study could provide additional evidence of race-specific genetic features in IRD and emphasize important roles of truncating variants, which lead to remnant protein functions for the pathogenesis of *USH2A*-RP.

## 2. Results

### 2.1. Syndromic and Non-Syndromic USH2A-RP

**Figure 1:**
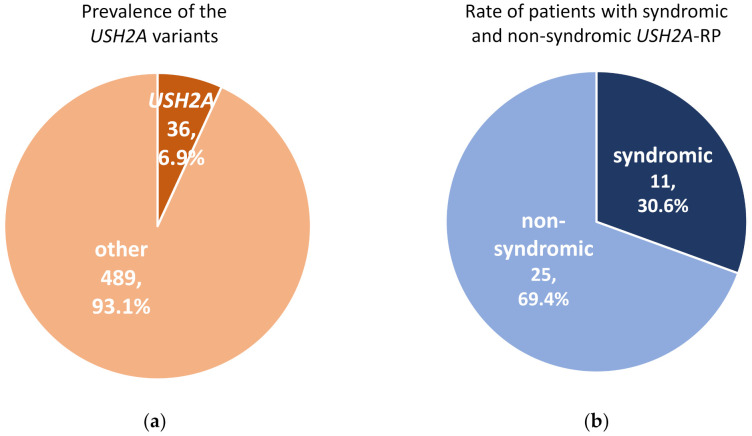
Prevalence of pathogenic variants in *USH2A* and rate of syndromic and non-syndromic *USH2A*-retinisis pigmentosa (RP) patients. (**a**) Pathogenic *USH2A* variants were detected in 36 of 525 RP cases. (**b**) There were 11 syndromic and 25 non-syndromic *USH2A*-RP patients in this study.

A total of 525 RP patients underwent genetic analysis and pathogenic variants were identified in 287 (54.7%) RP patients in this cohort. In these 525 RP patients, 36 (6.9%) cases carried *USH2A* variants which could explain their symptoms (Figure 1a). Among 36 patients with *USH2A*-RP, 11 (30.6%) patients were syndromic RP with congenital hearing loss (Figure 1b).

The genetic characteristics of syndromic and non-syndromic RP patients are presented in Table 1 and Table 2. Among both groups, no significant differences were observed regarding age, gender, and consanguineous marriage. All 36 *USH2A*-RP cases showed an autosomal recessive pattern of inheritance, but affected family members were found at a higher rate in syndromic *USH2A*-RP cases than non-syndromic *USH2A*-RP cases, although statistical differences were not detected (*p* = 0.25).

### 2.2. Truncating USH2A Variants were More Frequently Detected in Syndromic than Non-Syndromic USH2A-RP Patients

**Figure 2:**
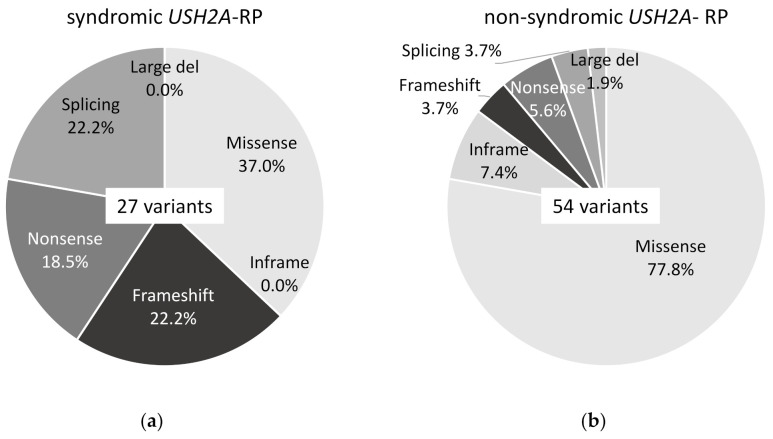
Frequency of each type of *USH2A* variant in this study. (**a**) Frequency of 6 types of 27 *USH2A* variants in syndromic *USH2A*-RP patients (*n* = 11). (**b**) Frequency of 6 types of 54 *USH2A* variants in non-syndromic *USH2A*-RP patients (*n* = 25).

Twenty-seven variants of *USH2A* were detected in 11 syndromic *USH2A*-RP patients and 54 variants in 25 non-syndromic *USH2A*-RP patients, which can explain their symptoms. Ten missense variants (37.0%), 6 frameshift variants (22.2%), 5 nonsense variants (18.5%), and 6 splicing variants (22.2%) were detected in syndromic *USH2A*-RP patients (Figure 2a). Forty-two missense variants (77.8%), 4 inframe variants (7.4%), 2 frameshift variants (3.7%), 3 nonsense variant (5.6%), 2 splicing variants (3.7%), and 1 large deletion variant (1.9%) were detected in non-syndromic *USH2A*-RP patients (Figure 2b).

Notably, a pattern of missense/missense or truncating/truncating was not observed in syndromic or in non-syndromic *USH2A*-RP patients, respectively (Table 3). In contrast to the observation that 32.0% (8 of 25) non-syndromic *USH2A*-RP patients had truncating variants such as nonsense, frameshift, or out of frame exon deletion, all syndromic RP patients (100.0%; 11 of 11) carried at least one truncating variant, which was significantly higher in syndromic RP (*p* < 0.01) (Table 3). All *USH2A*-RP patients with two truncating variants (*n* = 6) had both RP and early onset hearing loss.

Frequently detected *USH2A* variants were p.(Cys934Trp) (one syndromic patient and four non-syndromic patients), p.(Gly2752Arg) (five non-syndromic patients), and c.8559-2A>G (four syndromic patients and one non-syndromic patient) (Table 4).

**Figure 3:**
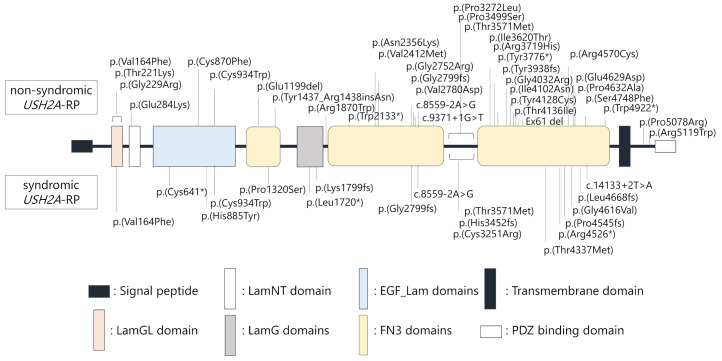
Schematic distribution of the *USH2A* variants identified in this study. Upper variants were detected in non-syndromic patients (*n* = 25), lower variants were detected in syndromic patients (*n* = 11).

Locations of each variant in the *USH2A* protein structure were schematically presented (Figure 3). Notably, *USH2A* variants were similarly found throughout entire regions of the *USH2A* protein structure in non-syndromic and syndromic *USH2A*-RP cases, suggesting that localization of variants does not correlate with hearing loss in *USH2A*-RP.

### 2.3. Earlier Onset of RP in Syndromic Patients than Non-Syndromic USH2A-RP Patients

**Figure 4:**
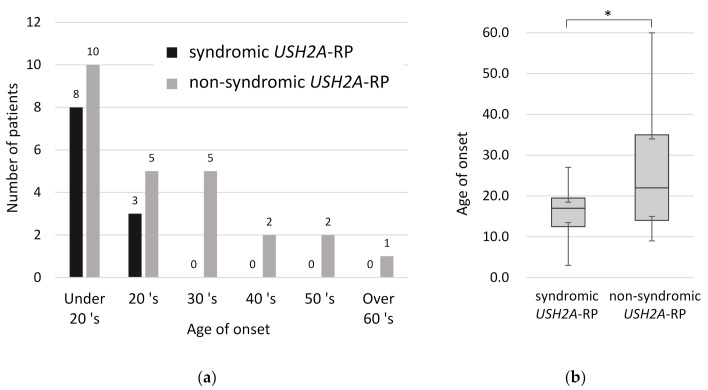
Onset age of RP in 11 syndromic and 25 non-syndromic *USH2A*-RP patients. (**a**) Distribution of onset age in syndromic and non-syndromic *USH2A*-RP patients. The vertical line shows the number of patients and the horizontal one shows the onset age of RP. Numeric numbers above each column indicate patients’ numbers. (**b**) Box plot of onset age in syndromic and non-syndromic *USH2A*-RP patients. The bottom and top of each box represent the lower and upper quartiles, respectively, and the line inside each box represents the median. The bottom and top bars represent the minimum and maximum value, respectively. *p* < 0.05 (*).

In order to understand clinical features between syndromic and non-syndromic *USH2A*-RP, age of disease onset was examined. All syndromic RP patients were aware of symptoms related to RP such as night blindness and constriction of visual field by their third decade of life (Figure 4a). Comparing the onset age of RP in 11 syndromic and 25 non-syndromic *USH2A*-RP patients, syndromic RP patients were aware of symptoms significantly earlier than non-syndromic patients (*p* < 0.05) (Figure 4b). The mean age of onset was 16.1 and 26.5 years old in syndromic and non- syndromic *USH2A*-RP patients, respectively.

### 2.4. Visual Acuity and Visual Field Constriction in Syndromic and Non-Syndromic USH2A-RP Patients

**Figure 5:**
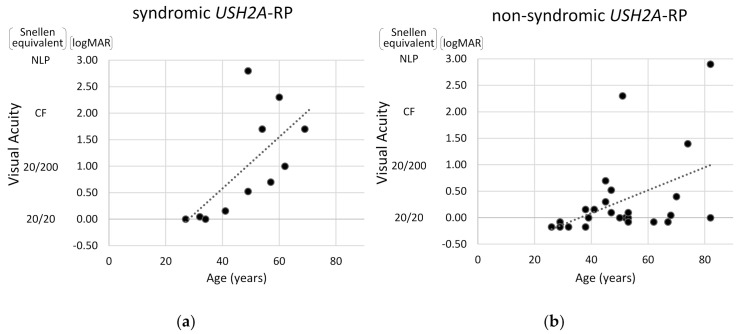
Scatter distribution of visual acuity and age in *USH2A*-RP cases. The vertical line shows visual acuity (Snellen equivalent and logMAR) and the horizontal one shows age of patient. One plot shows one patient and dash lines represent approximate straight lines. Results of 11 syndromic (**a**) and 25 non-syndromic *USH2A*-RP patients (**b**) are respectively presented. NLP: no light perception, CF: counting fingers.

Next, visual acuity and degrees of visual field constriction were compared between syndromic and non-syndromic *USH2A*-RP cases. As shown in Figure 5, the correlation between age and visual acuity was more pronounced in syndromic *USH2A*-RP patients than in non-syndromic *USH2A*-RP patients. These data also indicate that a decline in visual acuity is more rapid in syndromic *USH2A*-RP cases than non-syndromic *USH2A*-RP cases.

**Figure 6:**
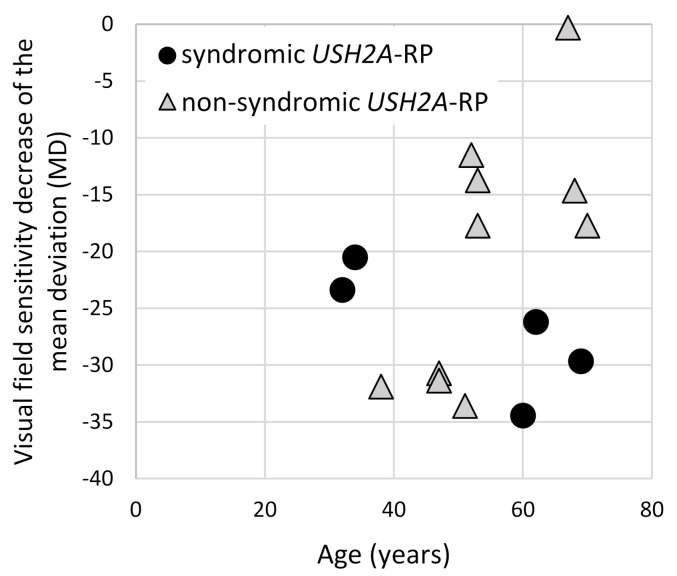
Correlation of the visual field changes and age of syndromic and non-syndromic *USH2A*-RP patients. The vertical line shows the mean deviation value (MD) which was obtained from HFA and the horizontal one shows age of patient. One dot represents each patient and round dots and triangle dots indicate syndromic (*n* = 5) and non-syndromic (*n* = 10) *USH2A*-RP patients, respectively.

To evaluate the visual field changes, HFA data were obtained from five syndromic and 10 non-syndromic *USH2A*-RP patients (Figure 6). Lower MD values and a decline in these values were revealed in syndromic *USH2A*-RP patients, indicating more severe visual field problems. Non-syndromic *USH2A*-RP cases were divided into two groups, a low MD (lower than −30) group aged in their 40s versus a moderate MD group in their 50s–70s. Because patients with truncating *USH2A*-variants belong to both groups, one of four in the low-MD group and two of six in the moderate-MD group, a clear contribution of truncating *USH2A* variants was not observed.

Additionally, time-dependent changes in the same patients were investigated. One syndromic and five non-syndromic *USH2A*-RP patients had performed HFA evaluations more than twice (Table 5). Rate of changes in MD values were −1.54 (dB/Y) in one syndromic *USH2A*-RP patient. On the other hand, the MD changes of −0.67 (dB/Y), −0.88 (dB/Y), −0.65 (dB/Y), −0.48 (dB/Y), and −0.34 (dB/Y) were observed in five non-syndromic *USH2A*-RP patients. These rates of MD changes in the syndromic *USH2A*-RP patient were higher than the average of the MD changes in non-syndromic *USH2A*-RP patients (−0.60 (dB/Y)).

Taken together, these results suggest that 1. truncating *USH2A* variants contribute to hearing loss in *USH2A*-RP cases and 2. syndromic *USH2A*-RP patients had a more severe retinal disease with earlier onset and a more rapid decline in visual function than non-syndromic *USH2A*-RP patients.

## 3. Discussion

In this study, genetic analyses of 525 RP patients were conducted using the targeted panel sequencing, and genetic and clinical features were characterized in 36 patients who were found to carry *USH2A* variants. Molecular diagnosis was made in 287 of 525 RP cases (54.7%) and *USH2A* disease-causing variants were identified in 6.9% of RP patients (36 of 525 patients) in this cohort. The frequency is similar with previous investigations in Japanese [2], reporting that *USH2A* is one of the major causal genes in Japanese RP patients.

All syndromic *USH2A*-RP patients in this cohort noticed their symptoms by their age of 30. The mean age at which our patients noticed symptoms, such as night blindness and visual constriction, was 16.1 years old in syndromic *USH2A*-RP patients and 26.5 years old in non-syndromic *USH2A*-RP patients, revealing that the onset of RP was significantly earlier in syndromic patients than in non-syndromic patients (*p* < 0.05). This trend has also been reported in previous reports of other ethnic groups [23]. In addition, not only age of onset but also RP symptoms were more severe in syndromic patients than in non-syndromic patients. A rapid decline in visual acuity was observed in syndromic *USH2A*-RP patients as compared to non-syndromic *USH2A*-RP patients whose acuity was reasonably well-maintained by their 70s. Although the data did not track changes in each patient over a long time, poorer visual acuity prognosis in syndromic *USH2A*-RP than non-syndromic *USH2A*-RP cases agreed with a trend in previous studies [22,23]. Similarly, as for visual acuity, syndromic *USH2A*-RP patients suffered more severe visual field constriction than non-syndromic *USH2A*-RP patients. In non-syndromic *USH2A*-RP patients, the rate of MD changes was approximately −0.5 (dB/year), which is the average reported progression rate of RP [27]. In contrast with non-syndromic *USH2A*-RP patients, one of our syndromic *USH2A*-RP patients showed the value of −1.5 (dB/year). In our clinic, the Goldmann perimetry is the first-choice visual field test for patients with low vision (lower than 1.0 of logMAR visual acuity), which was more preferably conducted in our syndromic *USH2A*-RP patients. Indeed 5 of 11 syndromic *USH2A*-RP patients had difficulty performing visual field tests with HFA due to their severely affected vision. These observations in visual acuity and visual field suggest that eye symptoms of *USH2A*-RP are more severe in patients with syndromic *USH2A*-RP than those with non-syndromic *USH2A*-RP.

Notably, the detection rate of truncating variants is significantly higher in syndromic *USH2A*-RP patients in this study. Considering characteristics of clinical features and genotype in *USH2A*-RP cases, truncating variants, which can largely affect protein functions and expression, possibly lead to severe symptoms related to RP and hearing loss. This genotype–phenotype correlation of variants which could contribute to remnant protein functions was reported in *CDHR1* [28], *ABCA4* [29], and *CDH23* [30,31]. Although syndromic RP cases without truncating variants in *USH2A* have been reported [23], truncating variants were detected in all syndromic *USH2A*-RP patients in this study, emphasizing the important roles of truncating variants for more severe phenotypes including hearing problems.

The variants frequently detected in this study were p.(Cys934Trp), p.(Gly2752Arg), and c.8559-2A>G. The last variant c.8559-2A>G is reported as a specific variant in the Japanese population [19]. On the contrary, *USH2A* variants often reported in the Caucasian population, such as p.(Cys759Phe) (pathogenicity of this variant is being reviewed elsewhere [32,33]) and p.(Glu767Serfs*21), were not identified in our Japanese cohort. Interestingly, the most frequent variant p.(Cys934Trp) was common in Chinese and Japanese populations, but other frequent variants differed. The variants often reported in the Chinese population, such as p.(Tyr2854_Arg2894del) and p.(Ser5060Pro), were not identified in our cohort. These observations suggest that there are unique variants in Japanese *USH2A*-RP patients as ethnic features.

It is not fully understood why *USH2A* variants lead to a wide range of phenotypes and severity of the diseases. *Ush2a*-knockout in mice and in zebrafish recapitulated a phenotype of human Usher syndrome with retinal degeneration and hearing problems [16,34]. These models could support our observation that all *USH2A*-RP patients with two truncating variants (n = 6) developed RP and early-onset hearing loss. Two protein isoforms, a long isoform and a short N-terminal isoform, are spliced from the *USH2A* gene [11]. Expression of the N-terminal isoform was only detected in the inner ear; in contrast, the long isoform was localized in photoreceptors and ears [16]. Interestingly, supplementation of a shortened form of *Ush2a* that lacks exon 12 rescued hearing loss in *Ush2a*-knockout mice [35]. Additionally, the roles of a partner protein, PDZD7, could be different in photoreceptors and inner ears [36]. Remarkably, *PDZD7* variants are only responsible for congenital hearing loss, but not for RP [36]. These facts and the accumulation of additional evidence could contribute to better understanding the pathogenesis of *USH2A*-associated diseases.

In our clinic, we recommend otorhinolaryngologic consultation for both syndromic and non-syndromic *USH2A*-RP patients to check their hearing ability. The previous study reported that some *USH2A*-RP patients are not aware of their hearing problems, suggesting non-syndromic *USH2A*-RP could have mild hearing deterioration [9]. Because we categorized groups of syndromic and non-syndromic *USH2A*-RP depending on the patients’ interview, possibilities of mis-grouping cannot be ruled out if such patients participated in this study. An additional concern could be that this study did not include patients who have only hearing loss due to *USH2A* variants. Pathogenic variants of *USH2A* in hearing loss have been reported in a previous meta-analysis [37]. Accordingly, further evaluations of *USH2A*-associated diseases from the standpoints both of ophthalmology and otolaryngology will be necessary.

In conclusion, truncating *USH2A* variants were more frequently identified in syndromic *USH2A*-RP patients who have congenital hearing loss than in non-syndromic *USH2A*-RP patients without hearing loss. Syndromic *USH2A*-RP patients have a more severe retinal disease with earlier-onset and a more rapid decline in visual function than non-syndromic *USH2A*-RP patients. Truncating variants could contribute to serious functional and tissue damages, suggesting important roles of truncating mutations for the pathogenesis of *USH2A*-RP, especially syndromic *USH2A*-RP.

## 4. Materials and Methods

### 4.1. Ethical Statement

All subjects gave their informed consent for inclusion before they participated in the study. The study was conducted in accordance with the Declaration of Helsinki, and the protocol was approved by the institutional Review Board of Kobe City Eye Hospital (Protocol no. E19002 and Permit no. ezh200901, 04.09.2020).

### 4.2. Patients Recruitment or Inclusion Criteria

A total of 525 IRD patients were included in this study. All the patients visited the IRD and Genetic Counseling Clinic in Kobe Eye Center Hospital from June 2015 to April 2020 (except four patients in 2013 and 2015) and gene alterations were identified in the genetic analysis studying IRD. In this cohort, patients with *USH2A* changes were further evaluated regarding their genetic and phenotypic characteristics.

### 4.3. Genetic Analysis

All patients underwent DNA sequencing using either a panel of 39 (238 patients) or 50 (287 patients) genes (Table 6) causing inherited retinal diseases which were selected based on previous reports [38,39,40]. *USH2A* (NM_206933.2) was included in both gene panels. The target capture panel covers entire coding exons and exon–intron boundaries of these genes, except *RPGR(ORF15)*. Targeted libraries were sequenced on an illumina NextSeq500 (NextSeq 500 System, illumina, San Diego, CA, USA).

The interpretation of sequence variants was performed based on the criteria and guidelines recommended by the American College of Medical Genetics and Genomics and the Association for Molecular Pathology [41]. Briefly, the variants shown below were classified as pathogenic variants: 1. null variants, which include nonsense, frameshift, start loss and out-of-frame exon deletion, and splice site (+/- 1,2); 2. variants with an allele frequency less than 5% in Exome Aggregation Consortium (ExAC), 1000 Genomes database, and Human Genetic Variation Databases (HGVD); 3. missense variants which were reported as disease-causing variants in previous reports or predicted a pathogenic effect in silico analysis (SIFT (https://sift.bii.a-star.edu.sg/) and PolyPhen2 (http://genetics.bwh.harvard.edu/pph2/index.shtml)). Clinvar information (https://www.ncbi.nlm.nih.gov/clinvar/) and previous reports have also been used for the interpretation of variants.

Supplemental sanger sequencing of *RPGR(ORF15)* was performed in male patients whose pathogenic variants were not detected in panel analysis. Segregation analysis using sanger sequencing was also performed in family members. After a data filtering and interpretation process for the detected variants, the variants were checked against clinical conditions and family history of each patient to determine molecular diagnosis in an expert meeting. More details on the analysis can be found in our previous reports [40].

Sequence variants are described in accordance with recommendations from the Human Genome Variation Society [42].

### 4.4. Clinical Evaluations

Symptoms and other clinical information such as age, gender, age of onset, family history, and the presence of hearing loss, were obtained from their medical and genetic counseling records. Ophthalmological evaluations were performed in the IRD clinic. Evaluations include best corrected visual acuity (BCVA) with the Snellen chart, slit-lamp biomicroscopy, dilated indirect ophthalmoscopy, ophthalmic imaging including fundus autofluorescence and retinal cross-section with OPTOS 200Tx and a SPECTRALIS_Spectral (Heidelberg Engineering, Heidelberg, Germany) domain optical coherence tomography (OCT) scanner, full-field electroretinogram (ERG), visual fields with Goldmann perimetry (GP) and Humphrey field analyzer (HFA).

### 4.5. Statistical Analysis

Statistical analysis was performed with R version 3.1.3 (R Core Team (2015). R: A language and environment for statistical computing. R Foundation for Statistical Computing, Vienna, Austria, http://www.R-project.org/). The Welch Two Sample *t*-test was used to compare age of onset in each group of syndromic and non-syndromic RP, and Fisher’s exact test was used to compare the characteristics of the genetic mutations in each group. *p*-values < 0.05 were considered statistically significant in a two-sided test. The value a of visual acuity (a) was converted into the value of Logarithm of the Minimum Angle of Resolution (logMAR) for statistical analysis (=log(1/a)). The hand motion value has a logMAR conversion of 2.30, the light sense value has a log MAR conversion of 2.80, and the no light sense value has a logMAR conversion of 2.90 [43,44]. Regarding the visual field condition, Mean Deviation (MD) values which obtain from HFA test were used for statistical analysis.

## Figures and Tables

**Table 1:** *USH2A* variants detected in RP patients in this study.

ID	Nucleotide Change	Protein Change	Zygosity
P1	c.490G>T;c.13631dupG	p.(Val164Phe);p.(Pro4545Serfs*17)	Het;Het
P2	c.1923T>A;c.3958C>T;c.5396delA	p.(Cys641*);p.(Pro1320Ser) ^1^;p.(Lys1799Serfs*18)	Het;Het;Het
P3	c.13576C>T;c.13847G>T	p.(Arg4526*);p.(Gly4616Val)	Homo;Homo
P4	c.2653C>T;c.9751T>C; c.13576C>T;c.13847G>T	p.(His885Tyr) ^2^; p.(Cys3251Arg) ^2^;p.(Arg4526*)^2^;p.(Gly4616Val)^2^	Het;Het; Het;Het
P5	c.8559-2A>G;c.14133+2T>A	p.(?);p.(?)	Het;Het
P6	c.8396delG	p.(Gly2799Valfs*31)	Homo
P7	c.10353_10356delTCAT;c.13010C>T	p.(His3452Glnfs*4);p.(Thr4337Met)	Het;Het
P8	c.2802T>G;c.5158delC	p.(Cys934Trp);p.(Leu1720*)	Het;Het
P9	c.8559-2A>G;c.10712C>T	p.(?);p.(Thr3571Met)	Het;Het
P10	c.8559-2A>G;c.14004delG	p.(?);p.(Leu4668Phefs*10)	Het;Het
P11	c.8559-2A>G	p.(?)	Homo
P12	c.10859T>C;c.11328T>G	p.(Ile3620Thr);p.(Tyr3776*)	Het;Het
P13	c.14243C>T	p.(Ser4748Phe)	Homo
P14	c.3596_3598delAAG;c.8254G>A	p.(Glu1199del);p.(Gly2752Arg)	Het;Het
P15	c.662C>A;c.7068T>G;c.7234G>A	p.(Thr221Lys) ^1^;p.(Asn2356Lys);p.(Val2412Met) ^1^	Het;Het;Het
P16	c.490G>T;c.3595_3597delGAA	p.(Val164Phe);p.(Glu1199del)	Het;Het
P17	c.10859T>C;c.14766G>A	p.(Ile3620Thr);p.(Trp4922*)	Het;Het
P18	c.8559-2A>G;c.14243C>T	p.(?);p.(Ser4748Phe)	Het;Het
P19	c.11156G>A;c.13010C>T	p.(Arg3719His);p.(Thr4337Met)	Het;Het
P20	c.2802T>G;c.13847G>T	p.(Cys934Trp);p.(Gly4616Val)	Het;Het
P21	c.(11712_12066)del;c.15233C>G	p.(?);p.(Pro5078Arg)	Het;Het
P22	c.8254G>A	p.(Gly2752Arg)	Homo
P23	c.850G>A;c.2802T>G	p.(Glu284Lys);p.(Cys934Trp)	Het;Het
P24	c.4310_4312dupATA;c.8254G>A	p.(Tyr1437_Arg1438insAsn);p.(Gly2752Arg)	Het;Het
P25	c.6399G>A;c.13887G>T	p.(Trp2133*);p.(Glu4629Asp)	Het;Het
P26	c.2802T>G;c.9815C>T	p.(Cys934Trp);p.(Pro3272Leu)	Het;Het
P27	c.14243C>T;c.15233C>G	p.(Ser4748Phe);p.(Pro5078Arg)	Het;Het
P28	c.9371+1G>T;c.12094G>A	p.(?);p.(Gly4032Arg)	Het;Het
P29	c.3596_3598delAAG;c.8254G>A;c.13894C>G	p.(Glu1199del);p.(Gly2752Arg);p.(Pro4632Ala)^1^	Het;Het;Het
P30	c.685G>C;c.13708C>T	p.(Gly229Arg);p.(Arg4570Cys)	Het;Het
P31	c.8254G>A;c.8396delG	p.(Gly2752Arg);p.(Gly2799Valfs*31)	Het;Het
P32	c.2802T>G;c.11811_11812delCT	p.(Cys934Trp);p.(Tyr3938Argfs*8)	Het;Het
P33	c.490G>T;c.12383A>G	p.(Val164Phe);p.(Tyr4128Cys)	Het;Het
P34	c.2609G>T;c.5608C>T; c.12305T>A;c.15355C>T	p.(Cys870Phe) ^1^;p.(Arg1870Trp); p.(Ile4102Asn) ^1^;p.(Arg5119Trp)^1^	Het;Het; Het;Het
P35	c.10495C>T;c.10712C>T	p.(Pro3499Ser);p.(Thr3571Met)	Het;Het
P36	c.8339T>A;c.12407C>T	p.(Val2780Asp);p.(Thr4136Ile)	Het;Het

P1–P11 are syndromic RP patients and they are indicated in gray. Pathogenicity of each variant needs further evaluation in the patients with more than three variants detected. ^1^ Novel variants those pathogenicity are suggested by in silico analysis. ^2^ These 4 variants were confirmed by segregation analysis.

**Table 2:** Characteristics of syndromic and non-syndromic RP patients.

Characteristics		Syndromic RP ^1^	Non-Syndromic RP ^2^
Age (years, mean ± SD, range)		48.5 ± 12.9, 27–69	50.9 ± 15.7, 26–82
Gender (*n*, %)	Male	6 (54.5)	13 (52.0)
Family History (*n*, %)	Yes	5 (45.5)	6 (24.0)
Consanguineous Marriage (*n*, %)	Yes	1 (9.1)	2 (8.0)

^1^ syndromic *USH2A*-RP patients (*n* = 11), ^2^ non-syndromic *USH2A*-RP patients (*n* = 25).

**Table 3:** Three types of combinations of *USH2A* variants in this study.

Type of Variant	Syndromic RP ^1^	Non-Syndromic RP ^2^
Truncating / Truncating (n, %)	6 (54.5)	0 (0.0)
Truncating / Missense (n, %)	5 (45.5)	8 (32.0)
Missense / Missense (n, %)	0 (0.0)	17 (68.0)

^1^ syndromic *USH2A*-RP patients (*n* = 11), ^2^ non-syndromic *USH2A*-RP patients (*n* = 25).

**Table 4:** *USH2A* variants detected in this study.

*USH2A* Variants	Syndromic RP ^1^	Non-Syndromic RP ^2^
p.(Val164Phe)	1	2
p.(Cys934Trp)	1	4
p.(Glu1199del)	0	3
p.(Gly2752Arg)	0	5
p.(Gly2799Valfs*31)	1	1
c.8559-2A>G	4	1
p.(Thr3571Met)	1	1
p.(Ile3620Thr)	0	2
p.(Ser4748Phe)	0	3
p.(Thr4337Met)	1	1
p.(Arg4526*)	2	0
p.(Gly4616Val)	2	1
p.(Pro5078Arg)	0	2

^1^ syndromic *USH2A*-RP patients (*n* = 11), ^2^ non-syndromic *USH2A*-RP patients (*n* = 25).

**Table 5:** Rate of changes in MD values of *USH2A*-RP patients.

Phenotype	Patient	Rate of Changes in MD Values	Observation Period (Years)
syndromic RP	P8	−1.54	1
non-syndromic RP	P12	−0.67	8
	P25	−0.88	1
	P27	−0.65	6
	P33	−0.48	2
	P36	−0.34 *	6

* The value of P36 was calculated with the left eye. P36 was not able to undergo HFA of the right eye due to low vision (hand motion) in the latest exam.

**Table 6:** The list of genes in our target capture panel.

*ABCA4*	*BEST1*	*BBS1* *	*C2orf71*	*CEP290* *
CDH23 *	CDHR1 *	CHM *	*CNGA1*	*CNGB1*
*CNGB3*	*CRB1*	*CRX*	*CYP4V2*	*EYS*
FAM161A *	GPR98 *	GUCA1A *	*GUCY2D*	*IMPDH1*
*IMPG2*	KLHL7 *	*LRAT*	*MAK*	*MERTK*
MYO7A *	*NR2E3*	*NRL*	PCDH15 *	*PDE6B*
*PRCD*	*PROM1*	*PRPF31*	*PRPF6*	*PRPH2*
*RDH5*	*RDH12*	*RHO*	*ROM1*	*RP1*
*RP1L1*	*RP2*	*RP9*	*RPE65*	*RPGR*
RS1 *	*SNRNP200*	*TOPORS*	*TULP1*	*USH2A*

* indicates genes involved only in the 50 genes panel.

## Abbreviations

RP	Retinitis Pigmentosa
IRD	Inherited Retinal Degenerative Disease
*USH2A*-RP	*USH2A*-associated RP
HFA	Humphrey field analyzer
MD	Mean Deviation
